# Ubiquitin-Specific Peptidase 8 Modulates Cell Proliferation and Induces Cell Cycle Arrest and Apoptosis in Breast Cancer by Stabilizing Estrogen Receptor Alpha

**DOI:** 10.1155/2023/8483325

**Published:** 2023-01-04

**Authors:** Lewei Zheng, Qian Yang, Chengxin Li, Gaoran Xu, Qianqian Yuan, Jinxuan Hou, Gaosong Wu

**Affiliations:** Department of Thyroid and Breast Surgery, Zhongnan Hospital of Wuhan University, 169 Donghu Road, Wuhan, Hubei 430071, China

## Abstract

Breast cancer (BC) is the most common neoplastic and lethal malignancy in women. Although antiendocrine therapy is the main treatment for estrogen receptor alpha (ER*α*)-positive BC, the development of resistance is a major clinical complication. In this study, we aimed to explore the role of ubiquitin-specific peptidase 8 (USP8) in ER*α* signaling and identify potential targets for endocrine resistance. Public databases were used to analyze USP8 expression, prognosis, clinical characteristics, and immune cell infiltration. Immunohistochemistry and western blot assays were used to detect protein levels and ER*α* signaling. Quantitative reverse transcription-PCR was used to measure ER*α* target gene expression. The cell counting kit-8, wound-healing, clone formation, and Transwell assays were used to investigate the effects of USP8 depletion or inhibition on cell proliferation, migration, and invasion. An immunofluorescence assay was used for localizing USP8 and ER*α*, and a protein stability assay was performed for detecting the degradation of ER*α* protein. The cell cycle and apoptosis were assessed using flow cytometry. USP8 was highly expressed in the luminal subtype of BC and was associated with poor prognosis. The infiltration levels of many immune cells were positively correlated with USP8 expression. Depletion of USP8 dramatically decreased the ER*α* signaling activity and weakened the proliferation, migration, and invasion capabilities of BC cells. USP8 knockdown markedly induced apoptosis and cell cycle arrest (*G*0/*G*1). Colocalization analysis and protein stability assays indicated a probable mechanism by which USP8 regulates ER*α*. Our study demonstrates that USP8 might be crucial in BC development and may be considered a potential target for treating ER-positive BC malignancies in vitro.

## 1. Introduction

Breast cancer (BC) is the most prevalent and lethal malignancy in women worldwide [1]. BC is classified into diverse clinical subsets, according to estrogen receptor alpha (ER*α*), progesterone receptor (PR), and human epidermal growth factor 2 (HER2) expression [2]. Among these types, ER-positive tumors, also referred to as luminal BC, are the predominant subtype, accounting for nearly 80% of cases [3]. Previous studies have indicated that most ER-positive BC patients can benefit from anti-ER*α* therapy; nevertheless, approximately half of them may develop drug resistance. ER*α* plays a crucial role in the development and progression of BC, as it contributes to the expression of oncogenic proteins and induces cell cycle progression [4]. Posttranslational modifications are dynamic biological processes involved in ER*α* stability, which may lead to the amplification of ER*α* signaling and tamoxifen resistance [5]. E3 ligases [6–8] appear to facilitate ER*α* signaling by stabilizing the ER*α* protein. Because ER*α* plays a significant role in the development of drug resistance in luminal BC [9], it is important to explore this mechanism in detail for better management of endocrine therapy.

Ubiquitination is a dynamic process coregulated by ubiquitinating enzymes and deubiquitinases (DUBs), which can reverse ubiquitin signals [10]. There are more than 100 human DUBs, which can be classified into seven families [11]. Ubiquitin-specific protease 8 (USP8), also referred to as ubiquitin isopeptidase Y (UBPY), belongs to the largest family of DUBs [12]. Mutated USP8 becomes hyperactivated, resulting in lung, cervical and gastric cancer aggravation, and poor prognosis [13–16]. However, how USP8 regulates cell proliferation in BC remains unclear.

Several studies have demonstrated that tumor-infiltrating immune cells (TICs) are strongly associated with BC progression [17]. Immune components are likely to provide evidence of an immunotherapy response in BC [18]. Studies have shown a correlation between cancer prognosis and degree of immune cell infiltration [19–21]. However, the biological influence of TICs in ER-positive BC still requires further investigation.

In our study, we aimed to explore the role of USP8 in ER*α* signaling and identify potential targets for endocrine resistance.

## 2. Materials and Methods

### 2.1. Public Data Retrieval and Bioinformatics

USP8 expression was analyzed using UALCAN (https://ualcan.path.uab.edu). Survival time and status were obtained from TCGA dataset (https://portal.gdc.com) for estimating BC prognosis. For Kaplan–Meier curves, *p*-values and hazard ratios with 95% confidence intervals were obtained using log-rank tests and univariate Cox proportional hazards regression. The characteristics of patients with BC from the UALCAN and cBioPortal (https://www.cbioportal.org/) online tools were listed, and the mRNA expression matrix of the cancer cells was acquired from the CCLE dataset (https://portals.broadinstitute.org/ccle) [22]. The analysis was performed using the R software package *ggplot2* (v4.1.3). Correlations between USP8 and ER*α* targets, apoptosis-related, and cell cycle-regulated genes are presented as scatter plots of TCGA data using Pearson's correlation analysis.

### 2.2. Gene Set Enrichment Analysis (GSEA) and Analysis of Immune Infiltration

The c2.cp.keggv7.2 gene and hallmark collections were obtained from the molecular signatures database (MSigDB) and analyzed using the GSEA via homonymous software. The significant gene sets conformed to the standards of “nominal (NOM) *p* value <0.05” and “false discovery rate (FDR) *q*-value <0.25.”

The proportion of TIC profiles across BC cases was evaluated using CIBERSORT [23]. Only cases with a *p* value <0.05 were selected for the follow-up analysis. The correlation between USP8 expression and tumor purity and immune cell infiltration levels in BC and luminal subtypes was investigated using the Tumor Immune Estimation Resource (TIMER) 2.0 platform (https://timer.comp-genomics.org/).

### 2.3. Cell Lines and Cell Culture

Cells were acquired from the American Type Culture Collection (ATCC), and human BC cell lines MCF7 and T47D were cultured in minimum essential medium and Roswell Park Memorial Institute-1640 medium (Biosharp, China) supplemented with 10% fetal bovine serum (FBS). HEK293T cells were grown in Dulbecco's Modified Eagle Medium supplemented with 10% FBS. All cells were grown at 37°C in a humidified 5% CO_2_ incubator. 17*β*-Estradiol (E2; Sigma-Aldrich) was dissolved in ethanol when required for the assays.

### 2.4. Immunohistochemistry (IHC)

BC specimens were obtained from the remaining tissues of patients who underwent surgery from September 2021 to March 2022 at the Zhongnan Hospital of Wuhan University. All diagnoses were confirmed by two experienced pathologists. ER, PR, HER2, and triple-negative BC (TNBC) subtypes were included. Specific primary antibodies against USP8 were used for IHC. Immunohistochemical scores were assessed using the ImageJ software.

### 2.5. Lentivirus Vector Construction

Full-length and deletion mutant constructs of the USP8 plasmid (UBPY and HumORF8) were obtained from Tsingke Biotechnology Co. Ltd. (Beijing, China). *Escherichia coli* DH5*α* cells were used for USP8 plasmid DNA amplification. The empty lentivirus vector LV-PURO-green fluorescence protein (GFP) was used as a control. The lentivirus was packaged using Opti-Mem, pMD2.G, PxpaX2, and overexpression plasmids and then used to infect 293T cells. Lentiviral small hairpin RNA (shRNA) virus-containing supernatants were used to infect MCF7 and T47D cells after 48 h. At 24 h postinfection, cells were sorted for GFP fluorescence. All USP8-silenced cells were selected with 0.5 *μ*g/mL puromycin. The USP8 shRNA sequences used were shRNA #1 (5′-GCTGTGTTACTAGCACTATAT-3′) and shRNA #2 (5′-CCTCACATCTAATGCTTACAA-3′).

### 2.6. RNA Extraction and Quantitative Reverse Transcription-PCR (qRT-PCR) Analysis

Total RNA was extracted from cancer cells using a HiPure Total RNA Mini Kit (Magen), according to the manufacturer's protocol. cDNA was synthesized using a cDNA reverse transcription kit (Abclonal). We used a total volume of 10 *μ*L, including 2 × SYBR Master Mix (Abclonal), template cDNA, and a mixture of each forward and reverse primer. Nuclease-free water was used to dilute components. The qRT-PCR was conducted in triplicate. Relative expression was normalized to that of ubiquitously expressed 36B4 and calculated using the ΔΔCT method. The primer sequences used are listed in Supplementary Table S1.

### 2.7. Western Blot (WB) Analysis

MCF7 and T47D cells were lysed using the RIPA extraction reagent (Servicebio) supplemented with protease and phosphatase inhibitors. Total protein was separated using 10% or 12.5% sodium dodecyl sulfate polyacrylamide gel electrophoresis and transferred to a 0.45 *μ*m polyvinylidene fluoride membrane (Millipore). The membrane was then successively blocked in Tris-buffered saline with 0.05% Tween-20 containing 5% skim milk for one hour, treated with primary antibodies overnight and secondary antibodies for an hour (Supplementary Tables S2 and S3). An enhanced chemiluminescence WB substrate was used to develop the blots.

### 2.8. Cell Counting Kit-8 (CCK-8) Assay for Cell Viability

BC cells were plated in clear-bottom 96-well plates and processed according to the manufacturer's instructions. CCK-8 solution was added to each well, and the absorbance at 450 nm was measured every 24 h.

### 2.9. Wound-Healing (WH) Assay

Monolayer-confluent human breast cells were wounded with a single pass of a 200 *μ*L pipette tip, and cell medium was replaced with 1% FBS. The ImageJ software was used to assess the WH rate with the following equation:

(1) $$\begin{array}{c} WH\;rate\left(\%\right)=\frac{\left(initial\;wound\;area-nonhealed\;area\right)}{initial\;wound\;area}\text{.} \end{array}$$

### 2.10. Colony Formation Assay

After incubation for 2 weeks, colonies were fixed with paraformaldehyde and stained with 0.1% crystal violet. Only colonies with more than 50 cells were counted. The colony formation rate was estimated as the number of colonies/number of seeded cells × 100.

### 2.11. Transwell Assay

Transwell assays were used to assess cell migration and invasion capacity. For the invasion assay, the upper chambers were coated with Matrigel (BD BioCoat, USA). Indicated cells resuspended in serum-deficient medium (200 *μ*L) were seeded into the upper chambers, whereas the bottom wells were filled with complete medium (600 *μ*L). After 24 h, the cells migrating through the membrane were fixed with 4% paraformaldehyde and stained with 0.1% crystal violet and then counted under a microscope.

### 2.12. Flow Cytometry Analysis

MCF7 and T47D cells were adjusted to a density of 1 × 10^6^ cells/mL, inoculated in 6-well plates, and incubated at 37°C and 5% CO_2_. Cells were harvested, washed with phosphate-buffered saline, and centrifuged at 300 × *g*. Following the manufacturers' protocol, we resuspended the cell pellets in 1 mL DNA staining solution and incubated them at 37°C for 30 min without light. For apoptosis analysis, the pellets were resuspended in a mixture of 5 *μ*L fluorescein isothiocyanate (FITC)/Annexin V and 10 *μ*L PI staining solution combined with 500 *μ*L 1 × binding buffer and incubated for 10 min. Cell cycle distribution and apoptosis were monitored using flow cytometry with a FACScan flow cytometer (Beckman, cat. #FC500, USA).

### 2.13. Protein Stability Assay

Cells were treated with 100 *μ*M cycloheximide (CHX, HY-12320, MedChemExpress) for 0, 4, 8, and 12 h to inhibit protein synthesis. For proteasome inhibition experiments, cells were treated with 10 *μ*M MG132 (HY-13259, MedChemExpress) for 8 h and then collected. The rate of ER*α* degradation was evaluated using WB analysis.

### 2.14. Cell Immunofluorescence Assay

For immunofluorescence analysis, MCF7 and T47D cells were attached to slides by gentle cytospin, followed by fixation with 4% paraformaldehyde. Next, the cell membrane was penetrated with 0.3% Triton X-100 for 1 h and blocked with normal goat serum for 1 h at 37°C. After adding the primary antibody against USP8 and ER*α* (1 : 200) at 4°C overnight, the cells were incubated with fluorescent dye-labeled secondary antibody at room temperature for 1 h and cultured with antifluorescence quenching sealing solution containing 4′,6-diamidino-2-phenylindole. A Nikon A + laser scanning confocal system was used for observations.

### 2.15. Statistical Analyses

Student's *t*-test, Pearson's correlation coefficient, and Cox regression analysis were used for comparisons. Statistical significance was set at *p* < 0.05.

## 3. Results

### 3.1. USP8 Is Associated with Poor Outcome of BC and with ER*α* and PR Protein Levels in Human BC Specimens

**Figure 1:**
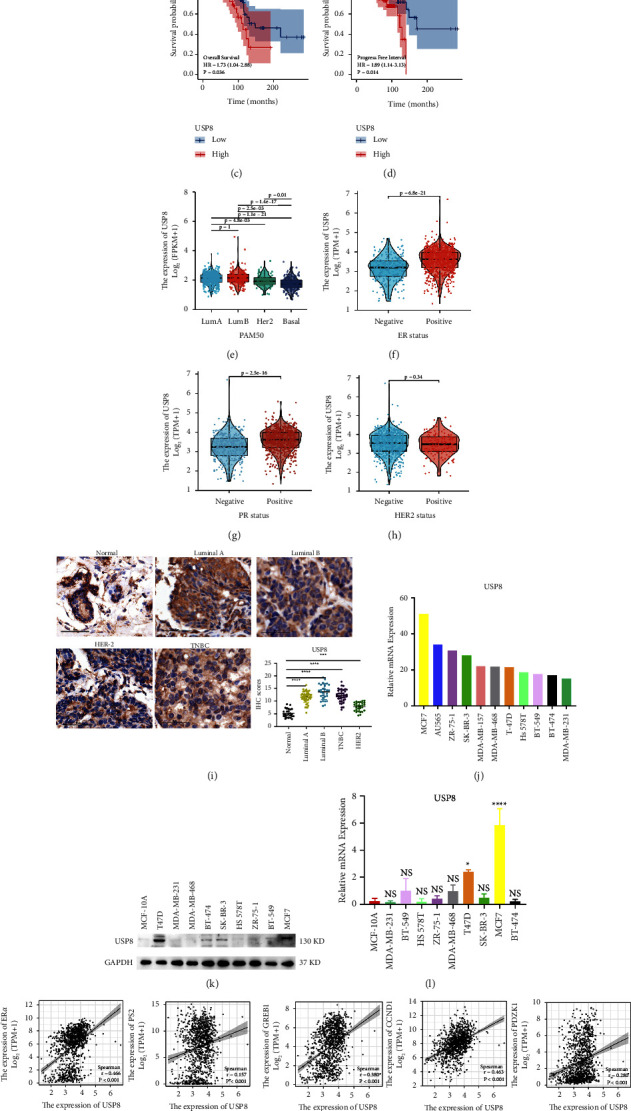
USP8 expression in samples and its correlation with survival, clinicopathological staging characteristics, and target gene expressions in BC patients. (a) USP8 upregulation in BC samples of CPTAC. (b–d) Downregulation of USP8 correlates with poor endocrine treatment outcomes in TCGA dataset. (e–h) The correlation of USP8 expression with clinicopathological characteristics. (i) Immunohistochemical results of patients with different subtypes of BC are shown at 100x magnification. (j) Expression levels of USP8 in different BC cells in the CCLE database. (k) Expression levels of USP8 in 10 BC cell lines. (l) mRNA expression levels of USP8 in 10 BC cell lines. (m) Pearson's correlation of USP8 expression with ER*α*, PS2, GREB1, CCND1, PDZK1, BAX, BCL2, CDK2, CDK4, and CDK6 expression.

USP8 is highly expressed in BC according to the CPTAC database (Figure 1(a)). The clinicopathological characteristics correlating with USP8 expression are shown in Table 1. USP8 expression showed significant differences according to race and the ER, PR, and PAM50 status. The survival Kaplan–Meier analysis of TCGA database revealed that USP8 is associated with poor prognosis in BC patients (Figures 1(b)–1(d)). In the BC cohort, USP8 expression was significantly associated with the ER and PR status, N stage, and PAM50 subtype but had no significant association with the HER2 status (Figures 1(e)–1(h)). Consistently, the level of USP8 protein was high in BC tissues, especially in ER-positive BC patients (Figure 1(i)). Based on the CCLE dataset, USP8 was also highly expressed in MCF7 and T47D BC cell lines (Figure 1(j)), consistent with our own finding (Figures 1(k) and 1(l)). As USP8 is highly expressed in the luminal BC subtype and is related to ER*α* protein levels, the correlation between USP8 and ER*α* target gene expression suggests that USP8 is positively associated with PS2, PDZK1, GREB1, and CCND1 (Figure 1(m)). Moreover, USP8 was also positively correlated with BCL2 and negatively correlated with BAX (Figure 1(m)). Furthermore, USP8 was positively associated with CDK2, CDK4, and CDK6 (Figure 1(m)).

### 3.2. Relationship between USP8 and Proportion of TIC Subtypes

**Figure 2:**
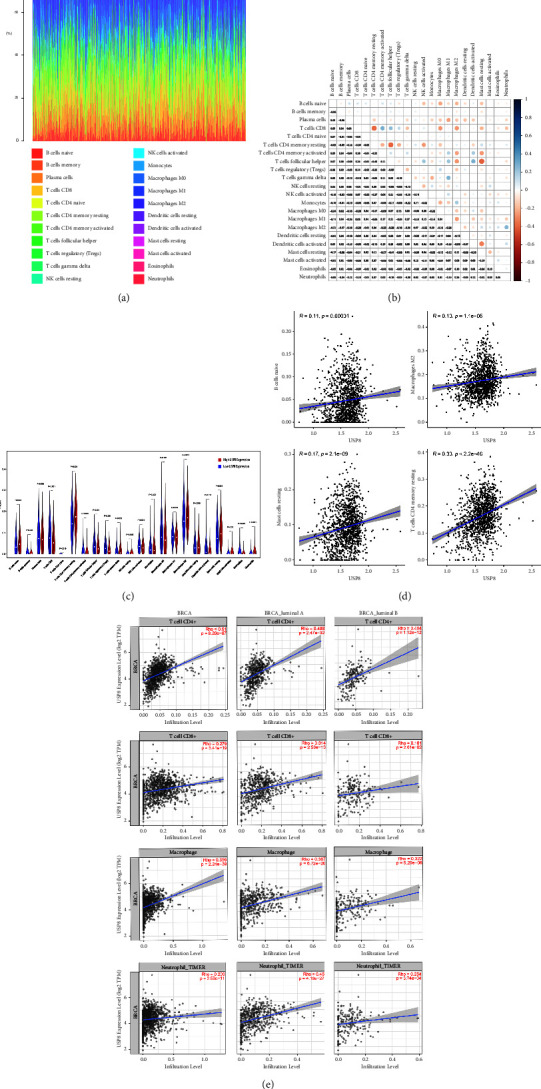
TIC profile in BC samples and correlation of the proportion of TICs with USP8 expression. (a) Bar plot showing the proportions of 22 types of TICs in BC samples. Column name = sample ID. (b) Heatmap showing the correlations between the 22 types of TICs. Numbers within boxes = *p* value of Pearson's correlation between two cells. The shadow of each box = correlation value between two cells. (c) Violin plot showing the ratio of differentiation of 22 TIC types between BC samples with low or high USP8 expression relative to the median of USP8 expression level. The Wilcoxon rank sum was applied for testing the significance. (d) Scatter plot of Pearson's correlations of four types of TICs with USP8 expression based on CIBERSORT (*p* < 0.05). The blue line = fitted linear model, indicating the proportion of the immune cell type along with USP8 expression. (e) Scatter plot showing Pearson's correlations of four types of TICs in different classifications of BC with USP8 expression based on TIMER 2.0 (*p* < 0.05).

Using the CIBERSORT method, we constructed the immune cell profiles of 22 BC cases and analyzed the proportion of TIC subtypes (Figures 2(a) and 2(b)). Four TIC subtypes, found to have a strong link with USP8 expression, were identified (Figures 2(c) and 2(d)). The infiltration levels of naive B cells, M2 macrophages, mast cells, and CD4+ T cells were positively correlated with USP8 expression. Analysis based on TIMER 2.0 also showed that the infiltration levels of most immune cells were positively associated with USP8 expression in both BC and luminal BC (Figure 2(e)).

### 3.3. Depletion of USP8 Inhibits Cell Proliferation, Migration, and Invasion in BC In Vitro

**Figure 3:**
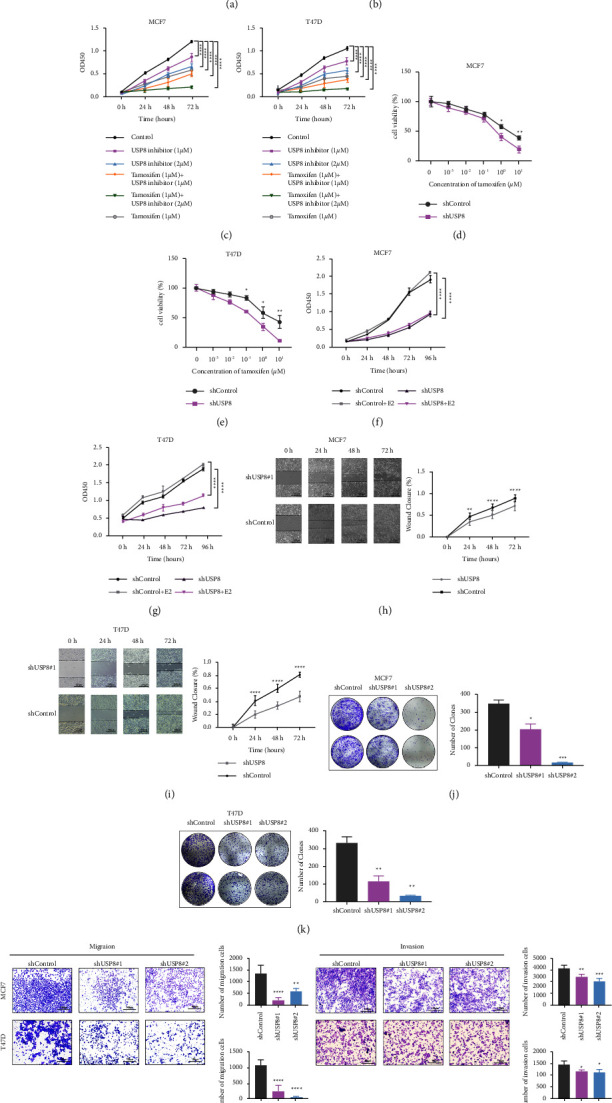
USP8 depletion or USP8 inhibitor inhibits BC cell proliferation and migration. (a) Inhibition of proliferation by DUB-IN-3 from 0 to 10 *μ*M. IC50 = 4.452 *μ*M. (b) Cell viability of MCF7 with 1 *μ*M or 2 *μ*M USP8 inhibitor. (c) Cell viability of MCF7 and T47D treated with 1 *μ*M or 2 *μ*M USP8 inhibitor in combination with 1 *μ*M tamoxifen. (d, e) USP8 depletion increased the sensitivity of MCF7 and T47D to tamoxifen. Cells were treated with the indicated concentration of tamoxifen. (f, g) USP8 depletion inhibited the proliferation of BC cells. (h, i) USP8 depletion inhibited the migration of BC cells. (j, k) USP8 depletion decreased the clone-formation capability of BC cells. (l) Transwell migration and invasion assay of BC cells.

To identify the effect of USP8 on BC phenotypes, MCF7 BC cell lines were treated with the USP8 inhibitor DUB-IN-3 in combination with tamoxifen. The half-maximal inhibitory concentration (IC50) was 4.452 *μ*M (Figure 3(a)). Overall, DUB-IN-3 reduced the proliferation rate of MCF-7 cells (Figure 3(b))—the higher the concentration, the more obvious the inhibition of cell proliferation, which was more apparent with the addition of tamoxifen (Figure 3(c)). Next, we used shRNA in BC cell lines. The knockdown efficiency of USP8 was verified at both the protein and transcriptional levels (Figure S1). USP8 knockdown also increased the sensitivity of MCF7 and T47D cells to tamoxifen (Figures 3(d) and 3(e)). The CCK-8 assay showed that USP8 depletion significantly inhibited the proliferation of MCF7 and T47D cells (Figures 3(f) and 3(g)). USP8 knockdown decreased the WH rate (Figures 3(h) and 3(i)), inhibited the clone formation capability (Figures 3(j) and 3(k)), and decreased the vertical migration and invasion capability of BC cells (Figure 3(l)).

### 3.4. Knockout of USP8 Impairs Cell Growth and Induces Apoptosis in Human BC Cells

**Figure 4:**
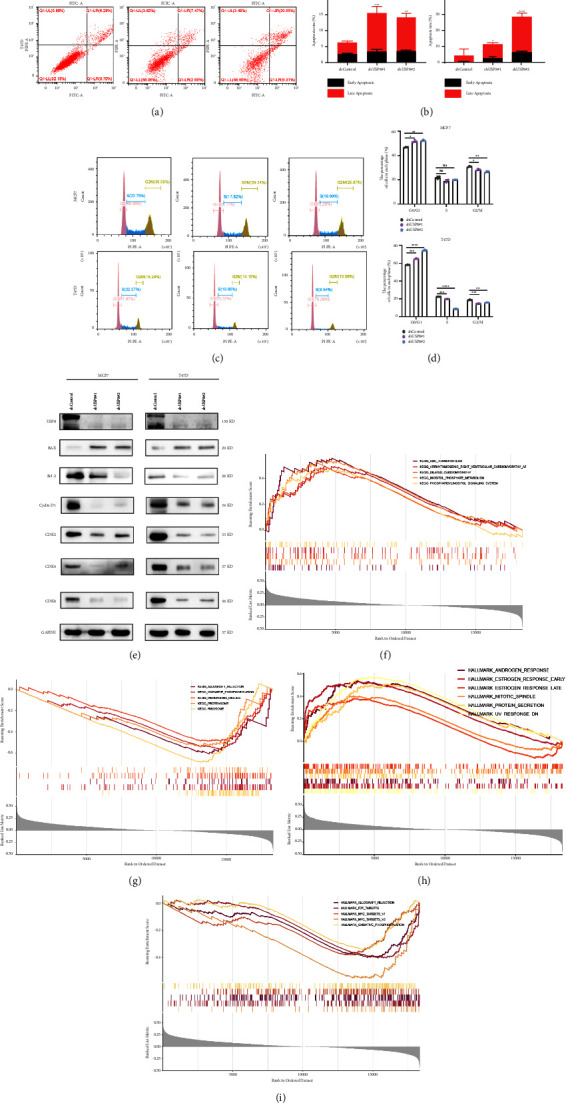
Induction of cell apoptosis and cell cycle arrest by USP8 knockdown and GSEA results of USP8. (a) Flow cytometry analysis of cell apoptosis in MCF7 and T47D treated with shUSP8 or shControl. PI PE-A stands for the fluorescence intensity of propidine iodide (PI), and FITC-A stands for the fluorescence intensity of fluorescein isothiocyanate (FITC)-labeled Annexin V. (b) Statistical analysis revealed the apoptotic rate (%) of both cell lines. (c) Flow cytometry analysis of the cell cycle in MCF7 and T47D treated with shUSP8 or shControl. Percentages (%) of cell populations at different stages of the cell cycle. (d) Statistical analysis of the percentages (%) of cell populations at different stages of the cell cycle in MCF7 and T47D treated with shUSP8 or shControl. (e) Immunoblot assay of apoptosis-related and cell cycle-related proteins in MCF7 and T47D treated with shUSP8 or shControl. (f) Enriched gene sets in the c2 KEGG gene collection in high-USP8-expression samples according to GSEA. Each line = *a* gene set; upregulated genes are located on the left, near the origin of the coordinates; downregulated genes are located on the right of the *x*-axis. Only gene sets with NOM *p* value <0.05 and FDR *q*-value <0.25 were considered significant. Only top gene sets are shown in the plot. (g) Enriched gene sets in the c2 KEGG gene collection in low-USP8-expression samples according to GSEA. (h) Enriched gene sets in the hallmark collection in high-USP8-expression samples according to GSEA. (i) Enriched gene sets in the hallmark collection in low-USP8-expression samples according to GSEA. ^*∗*^, *p* < 0.05; ^*∗∗*^, *p* < 0.01; ^*∗∗∗*^, *p* < 0.001.

Eligible cell models were constructed using shUSP8 in MCF7 and T47D cells. The knockdown efficiencies of shUSP8#1 and shUSP8#2 were evaluated using WB and flow cytometry analyses. The proportion of apoptotic cells increased upon shUSP8 treatment in both the cell lines (Figures 4(a) and 4(b)). USP8 depletion resulted in the downregulation of BCL2 and upregulation of BAX expression (Figure 4(e)). Cell cycle analysis revealed that USP8 knockdown considerably increased the ratio of cells in the G1 phase and the proportion of cells in the G2/M phase (Figures 4(c) and 4(d)). Consistent with the phenomenon of G1 arrest, the expression of cyclin D1, CDK2, CDK4, and CDK6 was repressed in shUSP8-treated cells (Figure 4(e)).

### 3.5. USP8 as a Putative Marker for Stabilizing ER*α*

GSEA was carried out individually for the high- and low-USP8-expression groups. For the c2 gene set in MSigDB, genes in the high-USP8-expression group were mainly enriched in metabolism-related biological processes (Figure 4(f)). Simultaneously, genes in the low-USP8-expression group were enriched in oxidative phosphorylation, protease, and ribosome-related processes (Figure 4(g)). Similarly, several immune activities and metabolic functions were enriched in the estrogen response in both early and late phases of hallmark gene sets (Figures 4(h) and 4(i)).

### 3.6. Depletion of USP8 Inhibits ER*α* Signaling Activity in BC

**Figure 5:**
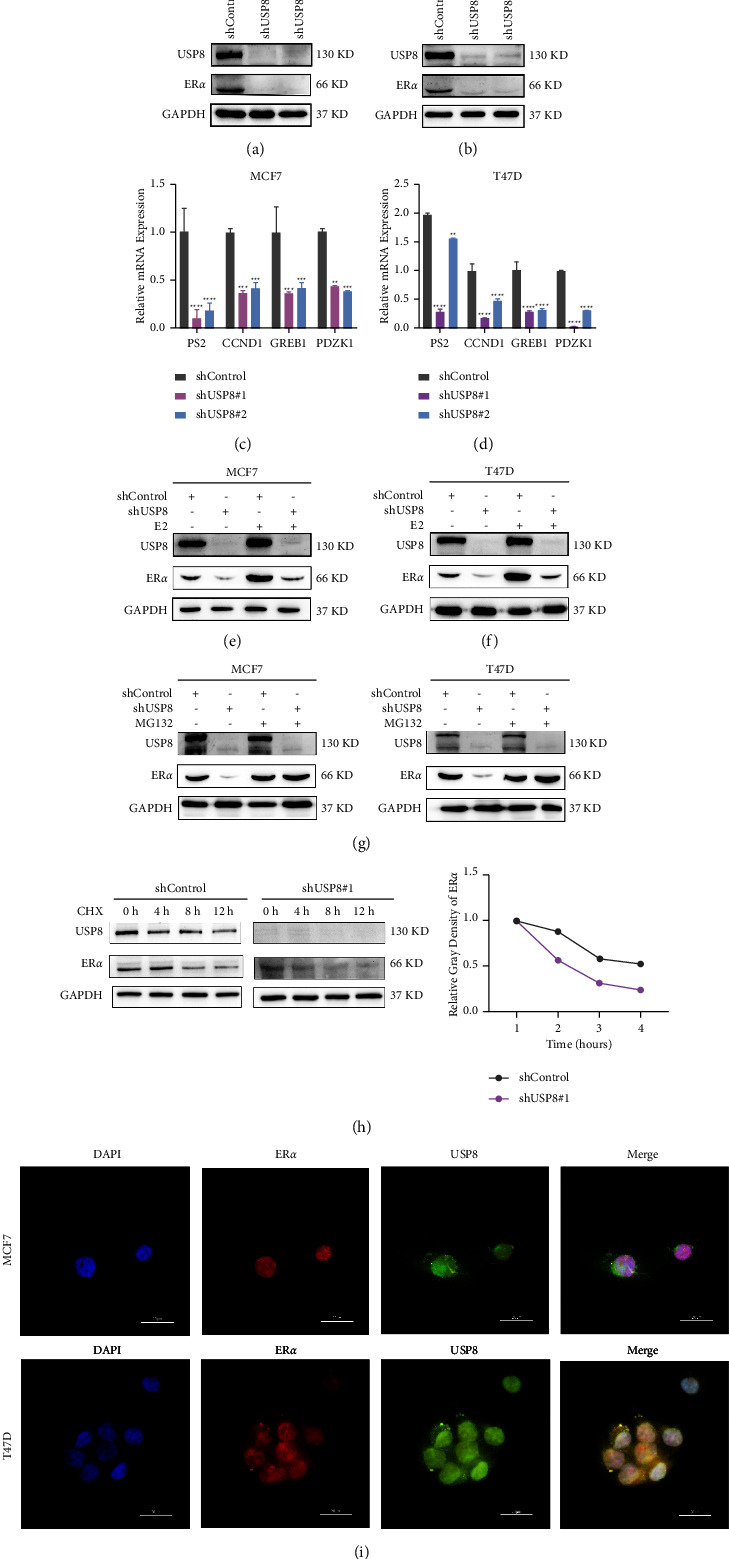
USP8 depletion promotes estrogen signaling activity. (a–d) USP8 knockdown decreased ER*α* target genes (*PS2*, *GREB1*, *CCND1*, and *PDZK1*) and protein levels. GAPDH was utilized as the internal control. (e, f) USP8 depletion effect on ER*α* protein level. Cells were treated with either ethanol or 10 nM estradiol for 6 h. (g) In the presence of the proteasome inhibitor MG132, shUSP8 did not further decrease ER*α* protein levels. Cells with shUSP8 or shControl were treated with 10 *μ*M MG132/vehicle for 6 h. The results are representative of three independent experiments. (h) USP8 depletion decreases ER *α*half-life in MCF7 and T47D cells. Cells were treated with 100 *μ*M CHX/vehicle for the indicated times. The relative density of protein bands was measured using the ImageJ software. (i) USP8 and ER*α* were partially colocalized in MCF7 and T47D cells (400 × magnification).

Compared with the shControl group, USP8 depletion markedly downregulated the expression of ER*α* protein and its target genes (*PS2*, *GREB1*, *CCND1*, and *IL-20*) (Figures 5(a)–5(d)). USP8 knockdown downregulated ER*α* protein levels and downstream target gene expression in both E2 and ethanol groups (Figures 5(e) and 5(f)).

### 3.7. USP8 May Interact with ER*α* in the Cytoplasm and Modulate ER*α* Protein Stability

An immunofluorescence assay showed that USP8 was localized to both the nucleus and the cytoplasm, whereas ER*α* was mainly localized to the nucleus, in BC cells (Figure 5(i)). USP8 knockdown significantly reduced the level of ER*α* protein, which could be reverted by the proteasome inhibitor MG132 (Figure 5(g)). As CHX inhibits protein synthesis, the protein half-life assay indicated that USP8 knockout in MCF-7 cells evidently impaired the endogenous stability of ER*α* (Figure 5(h)).

## 4. Discussion

The present results indicate that USP8 is upregulated in BC tissues and is associated with poor prognosis and immune cell infiltration. USP8 was localized to both the cytoplasm and nucleus, and its knockdown attenuated ER*α* signaling activity, cell proliferation, migration, and invasion, probably via ubiquitination of ER*α*. USP8 depletion stimulated cell cycle arrest and apoptosis.

ER-positive BC is characterized by slow disease progression and relatively good prognosis, while 30% of patients suffer from metastases or endocrine resistance due to clinical heterogeneity [24]. Various mechanisms have been proposed to elucidate endocrine resistance, including changes in ER regulators and different signaling pathways [25–29]. Posttranslational modifications have been studied for their role in ER*α* signaling, among which ubiquitination of ER*α* is a crucial factor in endocrine insensitivity [6–8, 30]. When ER*α* is stimulated with estrogen, it can be transferred to the nucleus and bind to *cis*-regulatory DNA region of target genes, promoting gene expression [31]. Tamoxifen, an estrogen receptor modulator, has tissue-specific agonistic and antagonistic effects on ER [32].

USPs are the most common DUBs; they can eliminate ubiquitin chains from the target proteins and may be involved in regulating the protein ubiquitination process [33]. USP8 has a catalytic domain located on its C-terminus and is upregulated in various malignancies [12]. We sought to elucidate the association between USP8 and ER signaling, as this mechanism remains unclear.

Our study shows that USP8 is highly expressed in BC samples in public databases and is associated with poor overall survival. USP8 was found to be upregulated in ER-positive BC compared with that in other subtypes, which lays the foundation for subsequent research.

Although anti-ER treatment is the primary therapy for ER-positive BC, immunotherapy cannot be neglected. Interestingly, while meaningful responses to immune modulation appear to be limited to TNBC [34], our findings on the positive relationship between USP8 expression and multiple immune cells indicate that USP8 is a potential target for immunotherapy. Dufner et al. regarded USP8 as an immunomodulatory DUB because mice with T cell-specific USP8 deficiency developed inflammatory bowel disease caused by its disrupting effect on regulatory T-cell functions [35]. A recent study showed that USP8 might be an immunomodulatory target that enhances the efficacy of anti-PD-1/PD-L1 in treating human carcinomas [36]. Compared with the existing work related to immune therapy in ER-positive BC patients [37], our study also shows that high USP8 expression may indicate better outcomes and responses to immune therapy due to the high proportions of TICs.

USP8 plays a vital role in potentiating cell proliferation in lung and cervical cancers [13, 15], leading to cell cycle dysregulation and accelerated apoptosis [16, 38, 39]. Similarly, our study confirms that depletion of both DUB-IN-3 and USP8 inhibited cell viability, which was inhibited more severely at higher concentrations of tamoxifen [40]. And tamoxifen sensitivity was further increased after USP8 knockdown. USP8 knockdown also suppressed the colony formation and migration capacity of BC cells. Furthermore, shUSP8 increased the apoptosis rate, with BAX (proapoptotic) upregulation and BCL2 (antiapoptotic) downregulation. USP8 depletion also caused cell cycle arrest at the G0/G1 phase in two different BC cell lines, which is in line with previous studies [39]. We further hypothesized that the cell cycle-related proteins CDK2/4/6 and cyclin D1 were reduced in the shUSP8 group.

We next observed that the depletion of USP8 decreased ER*α* protein levels. qRT-PCR and bioinformatics analyses showed that shUSP8 downregulated PS2, PDZK1, GREB1, and CCND1. WB assays demonstrated that USP8 stabilized ER*α*. When cells were treated with 10 nM E2 for 6 h, both USP8 and ER*α* protein levels were rescued (Figures 5(e)and 5(f)). Inhibition of the proteasomal degradation pathway with MG132 had a remarkable effect on stabilizing ER*α* protein levels even when USP8 was depleted. Based on the inhibitory effects of CHX on protein translation, we found that USP8 knockdown in MCF7 and T47D cells decreased the stability of endogenous ER*α*, which presented a shortened half-life in the shUSP8 group. Immunofluorescence analysis revealed that USP8 is localized to both the nucleus and cytoplasm, whereas ER*α* is mainly localized to the nucleus, indicating the location of interaction of both the proteins. In addition, we performed GSEA and found that high USP8 expression correlated with the estrogen response.

## 5. Conclusions

Overall, we verified the role of USP8 in ER-positive BC cells as an ER *α*stabilization-mediating deubiquitinase. USP8 expression is higher in ER-positive BC, and upregulation of USP8 mediates cell proliferation and apoptosis and facilitates the cell cycle of BC cells. We provide in vitro evidence that USP8 is a crucial mediator of endocrine resistance in ER-positive BC and could be a novel therapeutic target for treating endocrine-resistant cancers. Further research into the direct interaction between USP8 and ER*α* is warranted to determine the exact interaction between the two proteins.

## Figures and Tables

**Table 1:** Clinicopathological characteristics correlate with USP8 expression.

Characteristic	Levels	Low expression of USP8	High expression of USP8	*p* value
*N*		541	542	

*T* stage, *n* (%)	*T*1	127 (11.8%)	150 (13.9%)	0.377
	*T*2	324 (30%)	305 (28.2%)	
	*T*3	73 (6.8%)	66 (6.1%)	
	*T*4	16 (1.5%)	19 (1.8%)	

*N* stage, *n* (%)	*N*0	278 (26.1%)	236 (22.2%)	0.051
	*N*1	168 (15.8%)	190 (17.9%)	
	*N*2	49 (4.6%)	67 (6.3%)	
	*N*3	40 (3.8%)	36 (3.4%)	

*M* stage, *n* (%)	*M*0	440 (47.7%)	462 (50.1%)	0.443
	*M*1	12 (1.3%)	8 (0.9%)	

Pathologic stage, *n* (%)	Stage I	88 (8.3%)	93 (8.8%)	0.142
	Stage II	324 (30.6%)	295 (27.8%)	
	Stage III	110 (10.4%)	132 (12.5%)	
	Stage IV	12 (1.1%)	6 (0.6%)	

Race, *n* (%)	Asian	25 (2.5%)	35 (3.5%)	<0.001
	Black or African American	132 (13.3%)	49 (4.9%)	
	White	354 (35.6%)	399 (40.1%)	

Age, *n* (%)	≤60	307 (28.3%)	294 (27.1%)	0.443
	>60	234 (21.6%)	248 (22.9%)	

Histological type, *n* (%)	Infiltrating ductal carcinoma	386 (39.5%)	386 (39.5%)	1.000
	Infiltrating lobular carcinoma	103 (10.5%)	102 (10.4%)	

PR status, *n* (%)	Negative	219 (21.2%)	123 (11.9%)	<0.001
	Indeterminate	1 (0.1%)	3 (0.3%)	
	Positive	301 (29.1%)	387 (37.4%)	

ER status, *n* (%)	Negative	176 (17%)	64 (6.2%)	<0.001
	Indeterminate	0 (0%)	2 (0.2%)	
	Positive	346 (33.4%)	447 (43.2%)	

HER2 status, *n* (%)	Negative	270 (37.1%)	288 (39.6%)	0.340
	Indeterminate	8 (1.1%)	4 (0.6%)	
	Positive	82 (11.3%)	75 (10.3%)	

PAM50, *n* (%)	Normal	23 (2.1%)	17 (1.6%)	<0.001
	LumA	237 (21.9%)	325 (30%)	
	LumB	79 (7.3%)	125 (11.5%)	
	Her2	51 (4.7%)	31 (2.9%)	
	Basal	151 (13.9%)	44 (4.1%)	

Menopause status, *n* (%)	Pre	106 (10.9%)	123 (12.7%)	0.499
	Peri	17 (1.7%)	23 (2.4%)	
	Post	349 (35.9%)	354 (36.4%)	

Anatomic neoplasm subdivisions, *n* (%)	Left	282 (26%)	281 (25.9%)	0.975
	Right	259 (23.9%)	261 (24.1%)	

Radiation therapy, *n* (%)	No	221 (22.4%)	213 (21.6%)	1.000
	Yes	281 (28.5%)	272 (27.6%)	

OS event, *n* (%)	Alive	467 (43.1%)	464 (42.8%)	0.802
	Dead	74 (6.8%)	78 (7.2%)	

DSS event, *n* (%)	Alive	485 (45.6%)	493 (46.4%)	0.129
	Dead	50 (4.7%)	35 (3.3%)	

PFI event, *n* (%)	Alive	463 (42.8%)	473 (43.7%)	0.470
	Dead	78 (7.2%)	69 (6.4%)	

Age, median (IQR)		58 (48, 66)	58 (49, 68)	0.084

## Data Availability

All data supporting the results of this study are shown in this published article and supplementary documents.

## Abbreviations

BC: Breast cancer
ER*α*: Estrogen receptor alpha
USP8: Ubiquitin-specific peptidase 8
PR: Progesterone receptor
HER2: Human epidermal growth factor 2
DUBs: Deubiquitinases
UBPY: Ubiquitin isopeptidase Y
TICs: Tumor-infiltrating immune cells
GSEA: Gene set enrichment analysis
FDR: False discovery rate
TIMER: Tumor immune estimation resource
FBS: Fetal bovine serum
IHC: Immunohistochemistry
GFP: Green fluorescence protein
shRNA: Small hairpin RNA
WB: Western blot
CCK-8: Cell counting kit-8
FITC: Fluorescein isothiocyanate
CHX: Cycloheximide
WH: Wound-healing.

## Supplementary Materials

Supplementary Materials Table S1: primer sequence used for qRT-PCR. Table S2: list of primary antibodies. Table S3: list of secondary antibodies. Figure S1: knockdown efficiency of USP8.
