# Supplementary material for: Ubiquitin-Specific Peptidase 8 Modulates Cell Proliferation and Induces Cell Cycle Arrest and Apoptosis in Breast Cancer by Stabilizing Estrogen Receptor Alpha
Source: J Oncol. 2023 Jan 4;2023:8483325. doi: 10.1155/2023/8483325 (PMC9839415; doi:10.1155/2023/8483325)
Supplement: Supplementary Materials — Table S1: primer sequence used for qRT-PCR. Table S2: list of primary antibodies. Table S3: list of secondary antibodies. Figure S1: knockdown efficiency of USP8. [file 8483325.f1.zip › Supplementary Table S1 Primer sequence used for qRT.docx]

**Supplementary Table S1 Primer sequence used for qRT-PCR.**

| Target genes | | Human（5’ to 3’） |
| --- | --- | --- |
| USP8 | Forward | AAGGAGCAATCACAGCAAAGG |
|  | Reverse | CTGCATTCTTCGAGCATCCATTA |
| GREB1 | Forward | GGGATCTTGTGAGTAGCACTGT |
|  | Reverse | AATCGGTCCACCAATCCCAC |
| PS2 | Forward | GTCCCTCCAGAAGAGGAGTG |
|  | Reverse | AGCCGAGCTCTGGGACTAAT |
| CCND1 | Forward | GCTGCGAAGTGGAAACCATC |
|  | Reverse | CCTCCTTCTGCACACATTTGAA |
| PDZK1 | Forward | GCCAGGCTCATTCATCAAAGA |
|  | Reverse | CCTCTAGCCCAGCCAAGTCA |
| 36B4 | Forward | GGCGACCTGGAAGTCCAACT |
|  | Reverse | CCATCAGCACCACAGCCTTC |
