# Supplementary material for: Ubiquitin-Specific Peptidase 8 Modulates Cell Proliferation and Induces Cell Cycle Arrest and Apoptosis in Breast Cancer by Stabilizing Estrogen Receptor Alpha
Source: J Oncol. 2023 Jan 4;2023:8483325. doi: 10.1155/2023/8483325 (PMC9839415; doi:10.1155/2023/8483325)
Supplement: Supplementary Materials — Table S1: primer sequence used for qRT-PCR. Table S2: list of primary antibodies. Table S3: list of secondary antibodies. Figure S1: knockdown efficiency of USP8. [file 8483325.f1.zip › Supplementary Table S2 List of primary antibodies.docx]

**Supplementary Table S2 List of primary antibodies.**

| **Antigens** | **Name of antibody** | **Supplier** | **Species antibodies raised in** | **Dilution used** |
| --- | --- | --- | --- | --- |
| USP8 | UBPY (E-1)antibody | Santa Cruz sc-376130 | Mouse  Polyclonal antibody | 1:1000 (WB)  1:100 (IF) |
| ER | ER-alpha antibody | Affinity  #AF6058 | Rabbit  Polyclonal antibody | 1:1000 (WB)  1:100 (IF) |
| CDK2 | CDK2 (78B2) | Cell Signaling  Technology, #2546 | Rabbit  Monoclonal antibody | 1:1000 (WB) |
| CDK4 | CDK4 (D9G3E) | Cell Signaling  Technology, #12790 | Rabbit  Monoclonal antibody | 1:1000 (WB) |
| CDK6 | CDK6 (D4S8S) | Cell Signaling  Technology, #13331 | Rabbit  Monoclonal antibody | 1:1000 (WB) |
| Cyclin D1 | Cyclin D1 (92G2) | Cell Signaling  Technology, #2978 | Rabbit  Monoclonal antibody | 1:1000 (WB) |
| BAX | BAX antibody | Abclonal  A12009 | Rabbit  Polyclonal antibody | 1:1000 (WB) |
| Bcl-2 | Bcl-2 antibody | Abclonal  A11025 | Rabbit  Polyclonal antibody | 1:1000 (WB) |
| GAPDH | GAPDH antibody | Abclonal  AC027 | Rabbit  Polyclonal antibody | 1:10000 (WB) |
