# Supplementary material for: Ubiquitin-Specific Peptidase 8 Modulates Cell Proliferation and Induces Cell Cycle Arrest and Apoptosis in Breast Cancer by Stabilizing Estrogen Receptor Alpha
Source: J Oncol. 2023 Jan 4;2023:8483325. doi: 10.1155/2023/8483325 (PMC9839415; doi:10.1155/2023/8483325)
Supplement: Supplementary Materials — Table S1: primer sequence used for qRT-PCR. Table S2: list of primary antibodies. Table S3: list of secondary antibodies. Figure S1: knockdown efficiency of USP8. [file 8483325.f1.zip › Supplementary Table S3 List of secondary antibodies.docx]

**Supplementary Table S3 List of secondary antibodies.**

| **Secondary Detection System Used** | **Host** | **Dilution used** | **Supplier** |
| --- | --- | --- | --- |
| Anti-Rabbit IgG HRP | Goat | 1:10,000 (WB) | Affinity, China, Cat. #S0001 |
| Anti-Mouse IgG HRP | Goat | 1:10,000 (WB) | Affinity, China, Cat. #S0002 |
| Anti-Rabbit IgG (H+L) Fluor594-conjugated | Goat | 1:100 (IF) | Affinity, China, Cat. #S0006 |
| Anti-Mouse IgG (H+L) Fluor488-conjugated | Goat | 1:100 (IF) | Affinity, China, Cat. #S0017 |
| Hoechst 33342 (1 mg/ml) nucleic acid staining (DAPI) | - | 1:750 (IF) | Molecular Probes/Invitrogen, Carlsbad, CA, USA, cat. no. A11007 |
