# Supplementary figures and images for: Ubiquitin-Specific Peptidase 8 Modulates Cell Proliferation and Induces Cell Cycle Arrest and Apoptosis in Breast Cancer by Stabilizing Estrogen Receptor Alpha
Source: J Oncol. 2023 Jan 4;2023:8483325. doi: 10.1155/2023/8483325 (PMC9839415; doi:10.1155/2023/8483325)

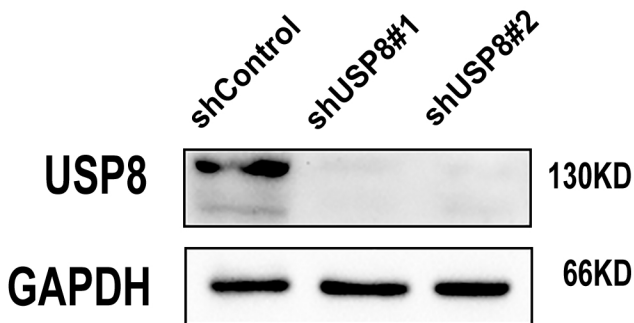

(a)

Supplement: Supplementary Materials — Table S1: primer sequence used for qRT-PCR. Table S2: list of primary antibodies. Table S3: list of secondary antibodies. Figure S1: knockdown efficiency of USP8. [file 8483325.f1.zip › FigS1-1.pdf]

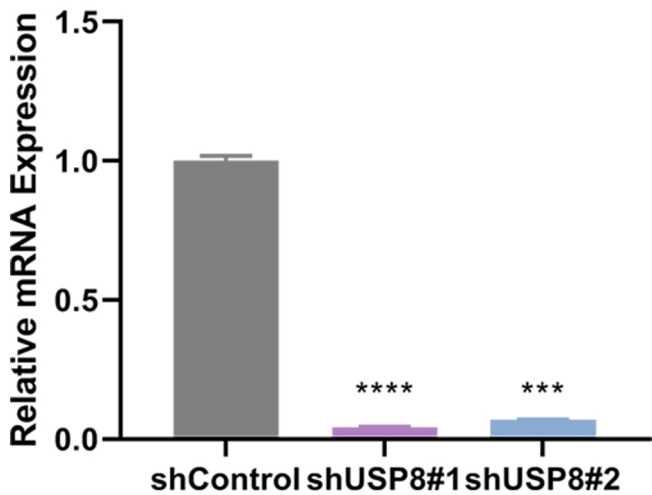

(b)

Supplement: Supplementary Materials — Table S1: primer sequence used for qRT-PCR. Table S2: list of primary antibodies. Table S3: list of secondary antibodies. Figure S1: knockdown efficiency of USP8. [file 8483325.f1.zip › FigS1-2.pdf]

Figure S1

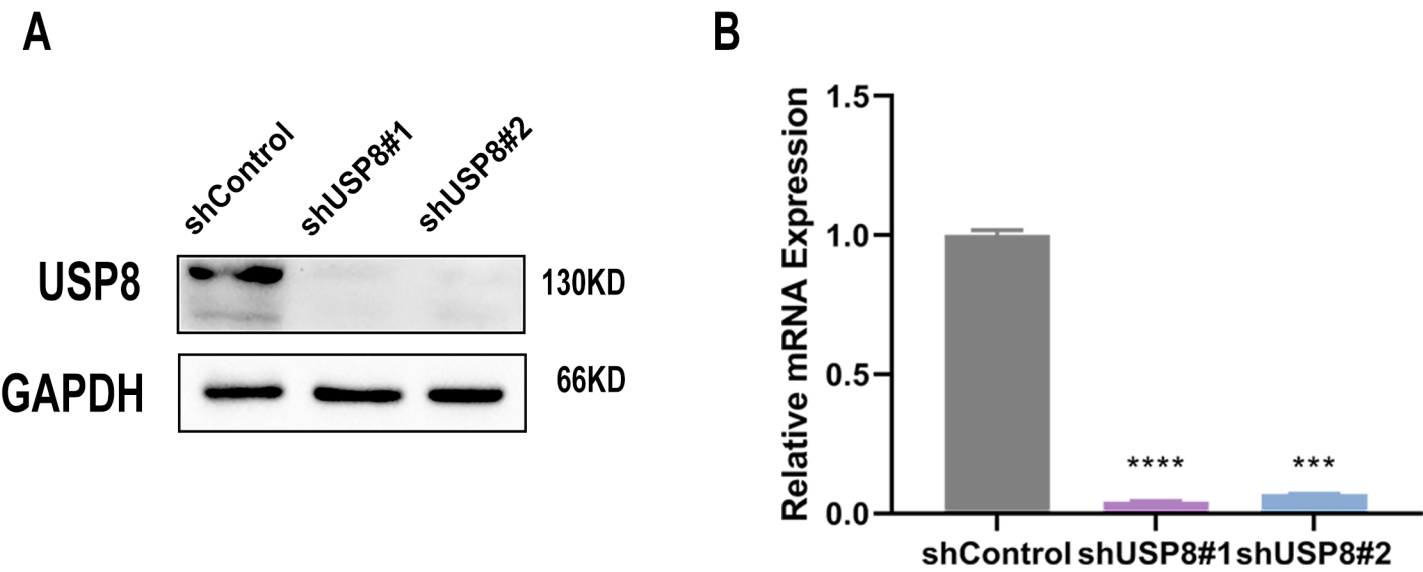

Supplement: Supplementary Materials — Table S1: primer sequence used for qRT-PCR. Table S2: list of primary antibodies. Table S3: list of secondary antibodies. Figure S1: knockdown efficiency of USP8. [file 8483325.f1.zip › Figure S1_original layout for reference (1).pdf]
